# The impact of COVID-19 on ischemic stroke

**DOI:** 10.1186/s13000-020-00994-0

**Published:** 2020-06-29

**Authors:** Pan Zhai, Yanbing Ding, Yiming Li

**Affiliations:** 1 Department of Neurology, Hubei Provincial Hospital of Tradition Chinese Medicine, Wuhan, 430073 Hubei China; 2 Department of Critical Care Medicine, Zhongnan Hospital of Wuhan University, 169 Donghu Road, Wuhan, 430071 Hubei China

**Keywords:** COVID-19, SARS-CoV-2, Ischemic stroke, Hypoxemia, Case report

## Abstract

**Background:**

The outbreak of a novel coronavirus since December 2019, became an emergency of major international concern. As of June 21, 2020, the SARS-CoV-2 pandemic has caused 8,769,844 confirmed infections with 463,745 fatal cases worldwide. The SARS-CoV-2 outbreak is a major challenge for clinicians. In our clinic, we found a rare case that a COVID-19 patient combined with ischemic stroke.

**Case presentation:**

A 79-year-old man was admitted to the Hubei Provincial Hospital of Traditional Chinese Medicine due to right limb weakness for 1 day and slight cough for 1 week. At presentation, his oxygen saturation was 94.2% on room air and body temperature was 37.3 °C (99.0 °F) with some moist rales. Neurological examination showed right limb weakness, and the limb muscle strength was grade 4. The left leg and arms were unaffected. In addition, runs of speech were not fluent enough with tongue deviation. Laboratory studies showed lymphopenia and eosinophilic granulocytopenia. Chest CT revealed bilateral pulmonary parenchymal ground-glass and consolidative pulmonary opacities, with a peripheral lung distribution. Real-time polymerase chain reaction (RT-PCR) from throat swab sample was positive for SARS-CoV-2 nucleic acid. This patient was treated with antiviral drugs and anti-inflammatory drugs with supportive care until his discharge. Clopidogrel (75 mg) and atorvastatin (20 mg) were administered orally to treat acute ischemic stroke. After 12 days of treatment, he can walk normally and communicate with near fluent language.

**Conclusion:**

We report an even more unusual case, a patient who was hospitalized for right limb weakness and was later diagnosed with COVID-19. Here, SARS-CoV-2 infection caused hypoxemia and excessive secretion of inflammatory cytokines, which contribute to the occurrence and development of ischemic stroke. Once COVID-19 patients show acute ischemic stroke, neurologists should cooperate with infectious disease doctors to help patients.

## Introduction

The emergence of severe acute respiratory syndrome coronavirus 2 (SARS-CoV-2; previously termed 2019 novel coronavirus or 2019-nCoV) disease at the end of 2019 has caused a large global outbreak and became a major public health issue. As of June 21, 2020, data from the World Health Organization (WHO) have shown that there is now a total of 8,769,844 reported cases of COVID-19 in 214 countries/regions, and 463,745 deaths. It is spread by human-to-human transmission via direct contact or aerosols [1]. The SARS-CoV-2 infection caused clusters of severe respiratory illness similar to severe acute respiratory syndrome coronavirus and was associated with ICU admission and high mortality [2]. Among patients with pneumonia caused by SARS-CoV-2, fever was the most common symptom, followed by cough [3]. Bilateral lung involvement with ground-glass opacity was the most common finding from computed tomography images of the chest [4]. Lymphopenia is a common feature in the patients with COVID-19 and might be a critical factor associated with disease severity and mortality [5].

The complications of COVID-19 included acute respiratory distress syndrome (29%), aemia (15%), acute cardiac injury (12%) and secondary infection (10%) [2]. 26.1% COVID-19 patients were transferred to the intensive care unit (ICU) because of complications [6]. In the course of clinical treatment of COVID-19, we found an interesting case that COVID-19 was followed by the onset of ischemic stroke, even though pneumonia is a frequent complication after stroke associated [7]. In this case report, we depicted a 79-year-old man who was hospitalized for right limb weakness and was later diagnosed with COVID-19. The relative roles of aging, oxidative stress, endothelial dysfunction, inflammation status, and other vascular risk factors could contribute to stroke [8, 9]. Hypoxemia and inflammation induced by COVID-19 may contribute to the occurrence, development and prognosis of ischemic stroke. This usual case suggests that neurologists should be vigilant in regards to COVID-19, especially those whose initial symptoms are neurological symptoms, and take effective measures of protection.

## Case presentation

**Fig. 1:**
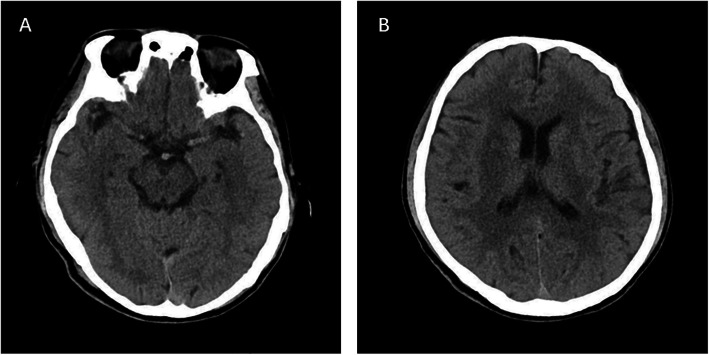
Brain CT images at different cross sections (**a** & **b**), which indicated lacunar cerebral infarction

On January 24, 2020, a 79-year-old man was admitted to the Hubei Provincial Hospital of Chinese Traditional Medicine (Guanggu, Wuhan, Hubei Province) due to right limb weakness for 1 day and slight cough for 1 week. At presentation, his body temperature was 37.3 °C (99.0 °F) with some moist rales. On January 7, 2020, he attended a large banquet and visited his friends. At administration, a neurological examination showed right limb weakness, and the limb muscle strength was grade 4. The left leg and arms were unaffected. In addition, runs of speech were not fluent enough with tongue deviation. The patient did not have nystagmus, oculomotor deficit, or sensory abnormality of the face, and the rest of the cranial nerve examination was normal. Deep tendon reflexes were symmetrically normal, cutaneous plantar response was flexor on both sides, and touch and vibration sense were normal. Medical, family, and psycho-social history were normal. The general examination indicated that the patient had hypertension (blood pressure: 165/120 mmHg). This patient did not have medical history of chronic hypertension. Brain CT showed lacunar infarction (Fig. 1). Forty-eight-hour Holter monitoring found paroxysmal atrial fibrillation.

**Fig. 2:**
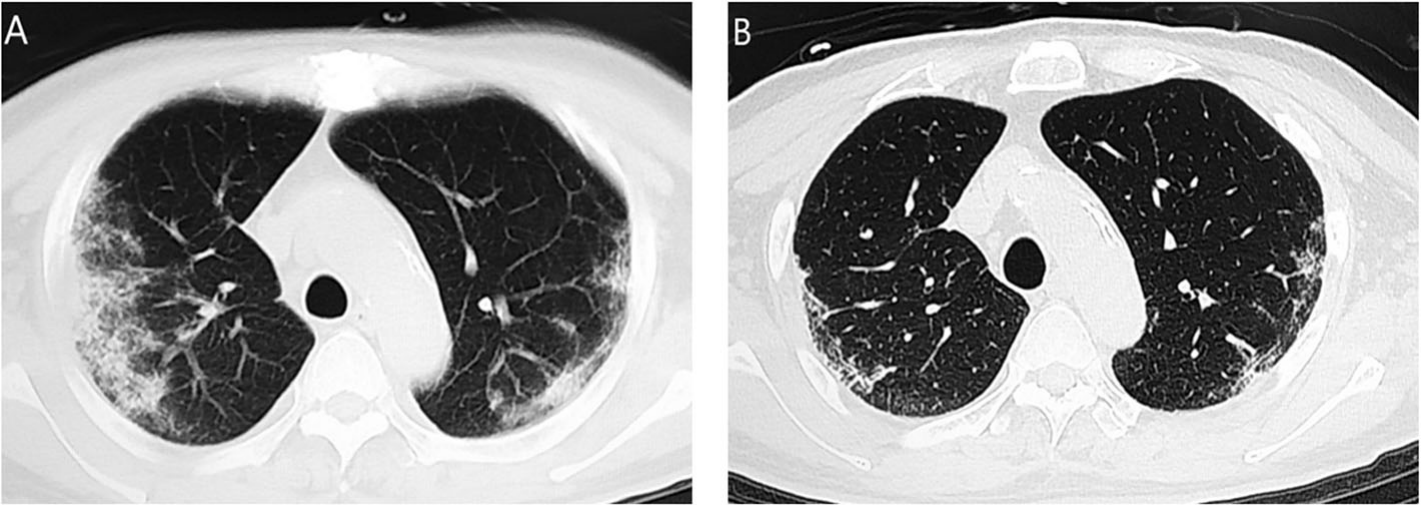
**a** CT scan obtained on the first day showed multifocal bilateral ground-glass opacities and consolidative pulmonary opacities. **b** CT scan obtained on day 12 showed that most of the lesions improved

Laboratory studies showed lymphopenia (lymphocyte cell count, 0.71 ×  10^9^/L) and eosinophilic granulocytopenia (eosinophilic cell count, 0.002 × 10^9^/L). The white blood cell count (7.08× 10^9^/L) was normal. The white blood cell differential count displayed 86.1% neutrophils (normal range, 40.0–70.0%), 6.0% lymphocytes (normal range, 20.0–50.0%), and 7.7% monocytes (normal range, 3.0–10.0%). The percentage of CD3+ T cells and CD3 + CD4+ helper T cells were decreased in the analysis of lymphocyte subsets. Several additional laboratory tests were abnormal, including C-reactive protein (36.1 mg/L; normal range, 0–10 mg/L), erythrocyte sedimentation rate (43 mm/h; normal range, < 20 mm/h), high density lipoprotein (1.04 mmol/L; normal range, 1.16–1.42 mmol/L), lipoprotein(a) (1276 mg/L; normal range, 10–300 mg/L), oxygen saturation (94.2%) on room air, and blood sugar (7.9 mmol/l; normal range, 3.9–6.1 mmol/l). The laboratory test results for cardiac, renal, and coagulation functions were normal. Since this patient noted having a slight cough, respiratory pathogens, including influenza virus, mycoplasma pneumoniae, adenovirus, coxsackievirus, and cytomegalovirus were checked, and these results were negative. The representative laboratory test results were shown in Table 1. Chest CT was performed at the same time, revealing bilateral pulmonary parenchymal ground-glass and consolidative pulmonary opacities, with a peripheral lung distribution (Fig. 2a). Real-time polymerase chain reaction from a throat swab sample was positive for SARS-CoV-2 nucleic acid.

**Table 1:** The representative laboratory test results

Test	Results	Nomal range
Lymphocyte cell count	0.71 × 10^9^/L	1.1–3.2× 10^9^/L
Eosinophilic cell count	0.002 × 10^9^/L	0.02–0.52× 10^9^/L
White blood cell count	7.08× 10^9^/L	3.5–9.5× 10^9^/L
Neutrophils percentage	86.1%	40.0–70.0%
Lymphocytes percentage	6.0%	20.0–50.0%
Monocytes percentage	7.7%	3.0–10.0%
CD3+ T cell percentage	45.0%	58.4–85.4%
CD3+ CD8+ T cell percentage	21.0%	19.17–39.7%
CD3+ CD4 + T cell percentage	24.0%	30–46%
CD19+ B cell percentage	19.0%	8.48–14.5%
CD16+ CD56+ NK cell percentage	37.0%	9.5–23.5%
C-reactive protein	36.1 mg/L	0–10 mg/L
Erythrocyte sedimentation rate	43 mm/h	< 20 mm/h
Procalcitonin	0.028 ng/ml	< 0.052 ng/ml
IL-6	16.49 pg/ml	< 7 pg/ml
High density lipoprotein	1.04 mmol/L	1.16–1.42 mmol/L
Lipoprotein(a)	1276 mg/L	10–300 mg/L
Blood sugar	7.9 mmol/L	3.9–6.1 mmol/L
ALT	37 U/L	9–50 U/L
AST	21 U/L	15–40 U/L
Respiratory syncytial virus IgM	Negative	Negative
Influenza A virus IgM	Negative	Negative
Influenza B virus IgM	Negative	Negative
Mycoplasma IgM	Negative	Negative
Adenovirus IgM	Negative	Negative
Coxsackievirus IgM	Negative	Negative

This patient was treated with antiviral drugs (oseltamivir, 75 mg, bid; ribavirin, 0.5 g, bid) and anti-inflammatory drugs (moxifloxacin, 0.4 g/day) with supportive care until his discharge on February 4, 2020. We prescribed five doses of dexamethasone (10 mg/day) until interim guidance from the WHO was released. Clopidogrel (75 mg) and atorvastatin (20 mg) were administered orally to treat acute ischemic stroke. Adverse and unanticipated events were not observed. The patient was not admitted to the ICU. After 12 days of treatment, he can walk normally and communicate with near fluent language. The respiratory symptoms of cough disappeared. Serial CT images illustrate the patient’s improvement after therapy (Fig. 2b).

The interest of the present case is 2-fold: (1) it is the first description of COVID-19 with right limb weakness as the initial symptom; and (2) it shows that COVID-19 is one of the pathogeneses of ischemic stroke.

## Discussion and conclusions

The outbreak of a novel coronavirus since December 12, 2019 become an emergency of major international concern, and  is a great challenge for clinicians. The typical symptoms are fever, sore throat, fatigue, cough or dyspnea coupled with recent exposure. The SARS-CoV-2 infection is of clustering onset, which is more likely to affect older males with comorbidities [10]. The elderly always have higher risk factors associated with acute ischemic stroke or embolization vascular events. Considering that this patient is 79 years old and the immune response is not as strong as that in young men, typical symptoms of SARS-CoV-2 infection were not observed. After he visited friends, 10 days later, he began to cough slightly. The mean incubation period of COVID-19 is 6.4 days, ranging from 2.1 to 11.1 days (2.5th to 97.5th percentile) [11]. We suspect that he was exposed to the viral environment, in which some aerosols may carry SARS-CoV-2. This patient was hospitalized with right limb weakness and disfluent speech, and chest CT examination later found typical images of COVID-19, suggesting that neurologists ought to inquire more information about the contact history. In addition, chest CT and laboratory examinations, including swab tests, should be prescribed for the diagnosis of COVID-19.

In this case, to explain right limb weakness as the initial symptom, it has been hypothesized that SARS-CoV-2 infection caused hypoxemia and excessive secretion of inflammatory cytokines, which induced acute ischemic stroke. Hypoxemia significantly reduces the energy required by cell metabolism, increases anaerobic fermentation, and causes intracellular acidosis and oxygen free radicals. With the continuation of hypoxia, intracellular calcium ion concentration increases significantly, inducing a series of cell damage, including apoptosis. Moreover, hypoxia can also induce inflammatory responses, including inflammatory cell infiltration and cytokine release, leading to further tissue ischemia [12]. Inflammation plays an important role in the occurrence, development and prognosis of cardiovascular and cerebrovascular diseases. Compared with non-ICU patients, ICU patients with a SARS-CoV-2 infection had higher plasma cytokine levels, including IL-2, IL-7, IL-10, GSCF, IP10, MCP1, MIP1A, and TNFα [2]. Inflammation contributes to atherosclerosis and affects plaque stability [13]. Additionally, the SARS-CoV-2 virus binds to angiotensin-converting enzyme 2 (ACE2) present on brain endothelial and smooth muscle cells. Depletion of ACE2 by SARS-CoV-2 may tip the balance in favor of the “harmful” ACE1/angiotensin II axis and promote tissue injury including stroke [14]. Following these ideas, we presume that SARS-CoV-2 infection pneumonia is one of the pathogeneses of ischemic stroke. The latest full-length genome sequences obtained revealed that SARS-CoV-2 uses the cell entry receptor ACE2 [15]. Structural analysis suggests that ACE2 can bind to B^0^AT1 (SLC6A19), which is a neutral amino acid transporter, providing important clues to the molecular basis for coronavirus recognition and infection [16]. Due to the combination of SARS-CoV-2 with the ACE2 receptor, some hypertensive patients with SARS-CoV-2 infection may experience abnormally elevated blood pressure, which increases the risk of intracerebral hemorrhage. In a familial cluster of pneumonia associated with SARS-CoV-2, older patients (aged > 60 years) have more systemic symptoms, such as thrombocytopenia, which enhances the chances of acute cerebrovascular events [5]. In the clinic, some COVID-19 patients have intracranial infection symptoms, including headache, epilepsy, and consciousness disorders; some COVID-19 patients have acute cerebrovascular disease symptoms, including paralysis of limbs; and a few have neuralgia and paresthesia.

Early diagnosis, quarantine, and supportive treatments are essential to cure patients. As SARS-CoV-2 is an emerging virus, no antiviral agents have been recommended for coronavirus infection [17]. In this case, low doses of dexamethasone therapy were used to avoid inflammatory storms. Corticosteroids suppress lung inflammation but also inhibit immune responses and pathogen clearance. The clinical outcomes of coronavirus and similar outbreaks do not support the use of corticosteroids [18–20]. Current interim guidance from the WHO on the clinical management of severe acute respiratory infection when SARS-CoV-2 infection is suspected (released January 28, 2020) advises against the use of corticosteroids unless indicated for another reason. For the treatment of hypertension, blockers of the AT1 receptor (ARBs), such as losartan, may be protective in stroke. While there has been some concern that ARBs and ACE inhibitors may be harmful in COVID-19 patients by increasing expression of ACE2 and SARS-CoV-2 binding, a joint statement from the American Heart Association. Under this controversial issue, amlodipine besylate tablets were given to lower blood pressure.

## Highlight

- We report an even more unusual case, a patient who was hospitalized for right limb weakness combined with poor speech fluency and was later diagnosed with COVID-19.
- During the period of the outbreak of COVID-19, neurologists should be vigilant in regards to COVID-19, especially those whose initial symptoms are neurological symptoms, and take effective measures of protection.
- COVID-19 may contribute to the occurrence, development and prognosis of ischemic stroke.
- Once COVID-19 patients show acute ischemic stroke, neurologists should cooperate with infectious disease doctors to help patients.

## Data Availability

The datasets used during the current study are available from the corresponding author on reasonable request.

## Abbreviations

COVID-19: Corona Virus Disease 2019
SARS-CoV-2: Severe acute respiratory syndrome coronavirus 2
ICU: Intensive care unit
